# Noninvasive Evaluation of GIP Effects on β-Cell Mass Under High-Fat Diet

**DOI:** 10.3389/fendo.2022.921125

**Published:** 2022-07-12

**Authors:** Sakura Kiyobayashi, Takaaki Murakami, Norio Harada, Hiroyuki Fujimoto, Yuki Murata, Naotaka Fujita, Keita Hamamatsu, Eri Ikeguchi-Ogura, Tomonobu Hatoko, Xuejing Lu, Shunsuke Yamane, Nobuya Inagaki

**Affiliations:** ^1^ Department of Diabetes, Endocrinology and Nutrition, Graduate School of Medicine, Kyoto University, Kyoto, Japan; ^2^ Radioisotope Research Center, Agency of Health, Safety and Environment, Kyoto University, Kyoto, Japan

**Keywords:** beta cell mass, GIP, exendin, SPECT, high-fat diet

## Abstract

Pancreatic β-cell mass (BCM) has an importance in the pathophysiology of diabetes mellitus. Recently, glucagon-like peptide-1 receptor (GLP-1R)-targeted imaging has emerged as a promising tool for BCM evaluation. While glucose-dependent insulinotropic polypeptide/gastric inhibitory polypeptide (GIP) is known to be involved in high-fat diet (HFD)-induced obesity, the effect of GIP on BCM is still controversial. In this study, we investigated indium 111 (^111^In)-labeled exendin-4 derivative ([Lys^12^(^111^In-BnDTPA-Ahx)]exendin-4) single-photon emission computed tomography/computed tomography (SPECT/CT) as a tool for evaluation of longitudinal BCM changes in HFD-induced obese mice, at the same time we also investigated the effects of GIP on BCM in response to HFD using GIP-knockout (GIP^-/-^) mice. ^111^In-exendin-4 SPECT/CT was able to distinguish control-fat diet (CFD)-fed mice from HFD-fed mice and the pancreatic uptake values replicated the BCM measured by conventional histological methods. Furthermore, BCM expansions in HFD-fed mice were demonstrated by time-course changes of the pancreatic uptake values. Additionally, ^111^In-exendin-4 SPECT/CT demonstrated the distinct changes in BCM between HFD-fed GIP^-/-^ (GIP^-/-^+HFD) and wild-type (WT+HFD) mice; the pancreatic uptake values of GIP^-/-^+HFD mice became significantly lower than those of WT+HFD mice. The different changes in the pancreatic uptake values between the two groups preceded those in fat accumulation and insulin resistance. Taken together with the finding of increased β-cell apoptosis in GIP^-/-^+HFD mice compared with WT+HFD mice, these data indicated that GIP has preferable effects on BCM under HFD. Therefore, ^111^In-exendin-4 SPECT/CT can be useful for evaluating increasing BCM and the role of GIP in BCM changes under HFD conditions.

## Introduction

Pancreatic β-cell mass (BCM) plays an important role in the pathogenesis of type 2 diabetes (1); preservation and expansion of BCM in response to metabolic demands thus represents a key strategy for preventing and treating diabetes. Expansion of BCM is induced by obesity and several other conditions or interventions including pregnancy (2–4), partial pancreatectomy (5), and administration of insulin receptor antagonist S961 (6). Obese individuals without diabetes also exhibit increased BCM (7), and BCM positively correlates with body mass index in nondiabetic Caucasians (8). However, previous findings on BCM were based only on cross-sectional studies using autopsy or surgically-resected pancreases; there is therefore a need to develop a noninvasive method for detection of changes in BCM (9). Recently, pancreatic β-cell-specific imaging using nuclear medicine techniques has been developed (10). In particular, exendin-based glucagon-like peptide-1 receptor (GLP-1R)-targeted imaging has emerged as a promising tool for noninvasive evaluation of BCM (9, 11–15). An indium 111 (^111^In)-labeled exendin-4 derivative ([Lys^12^(^111^In-BnDTPA-Ahx)]exendin-4) targeting GLP-1R has been developed (9, 16) that enables longitudinal analysis of BCM changes in living mice by quantification of pancreatic uptake of ^111^In-exendin-4 on single-photon emission computed tomography/computed tomography (SPECT/CT) images (9, 17). Several studies using ^111^In-exendin-4 SPECT/CT have demonstrated longitudinal decreases in BCM in NOD and db/db mice (18–21); however, *in-vivo* BCM expansion under obesity has not been examined longitudinally using noninvasive GLP-1R targeted imaging techniques.

To explore a potential of this unique approach to measure BCM using gain- and loss-of –function models, we employed HFD-fed and GIP-knockout (GIP^-/-^) mice. GLP-1 and glucose-dependent insulinotropic polypeptide/gastric inhibitory polypeptide (GIP) are the two incretins secreted from the intestine in response to meal ingestion, and promote glucose-dependent stimulation of insulin secretion *via* GLP-1 and GIP receptors on pancreatic β-cells (22, 23). GIP is synthesized in K cells, which are distributed mainly in the upper small intestine (24). Although GIP secretion is increased in obesity (25–27), the effect of GIP on *in-vivo* BCM remains controversial (28–31). In one report, the β-cell area in GIP receptor-knockout (GIPR^-/-^) mice was found to be increased compared to that in wild-type (WT) mice on a normal diet (28). In another report, the β-cell area in β-cell-specific GIP receptor-knockout (GIPR^-/-βCell^) mice was found to be decreased compared to that in control mice on normal diet (29). Furthermore, the β-cell area was decreased in GIPR^-/-^ mice on high-fat diet (HFD) as compared to that in WT mice (30), while the β-cell area of GIPR^-/-βCell^ mice was increased (29). In a report on GIP-knockout (GIP^-/-^) mice, β-cell area was decreased in GIP^-/-^ mice fed HFD as compared with that in WT mice (31). Importantly, these findings were based on conventional histological methods with restricted sampling in resected pancreas for cross-sectional BCM evaluation. Moreover, the longitudinal changes in BCM *in-vivo* in response to GIP deficiency could not be assessed.

In this study, we examined the utility of ^111^In-exendin-4 SPECT/CT in measuring longitudinal changes in BCM in mice developing obesity on HFD and GIP deficiency. In addition, we compared longitudinal *in-vivo* BCM changes in GIP^-/-^ and WT mice with and without HFD condition using ^111^In-exendin-4 SPECT/CT to assess the effects of GIP.

## Materials and Methods

### Animals

GIP-GFP knock-in mice were generated as described previously (32). This mouse exhibits no GIP secretion in the homozygous state (31, 33). Male GIP^-/-^ and littermate wild-type (WT) mice were maintained in a 14:10-h light-dark cycle with free access to water and food. WT and GIP^-/-^ mice were divided into the following two groups at 6 weeks of age: control-fat (CF; 10% fat by energy, 3.85 kcal/g) diet (WT+CFD, GIP^-/-^+CFD) and high-fat (HF; 45% fat by energy, 4.73 kcal/g) (Research Diets, New Brunswick, NJ) diet (WT+HFD, GIP^-/-^+HFD). Weekly body weight and energy intake were assessed during the observation period. Blood samples under non-fasting conditions, oral glucose tolerance test (OGTT), insulin tolerance test (ITT), and body fat composition analyzed by CT were performed at 6, 10, and 16 weeks of age. After 10 weeks on CFD or HFD, resected pancreatic tissue was harvested for immunohistochemical analysis. In addition, ^111^In-exendin-4 SPECT/CT scans were performed at 6, 10, and 16 weeks of age. This animal study was approved by the animal care and use committee of Kyoto University Graduate School of Medicine (MedKyo 19245, 19246, 21508).

### Blood Samples

Non-fasting blood samples (50 μL) were collected from the tail vein of mice at 10:00 AM at 6, 10, and 16 weeks of age. OGTTs were performed after a 16-hour fasting period. Blood samples for total GIP levels were collected *via* tail vein at 0 and 15 minutes; blood samples for blood glucose and plasma insulin were collected *via* tail vein at 0, 15, 30, 60, and 120 minutes after oral glucose administration (2 g glucose/kg body weight). Blood glucose levels were measured using the glucose oxidase method (Sanwa Kagaku Kenkyusho, Nagoya, Japan). Total GIP was measured with total GIP ELISA kit (Millipore, Billerica, MA, USA); plasma insulin was measured with an insulin ELISA kit (Shibayagi, Gunma, Japan). For ITTs, human regular insulin (100 U/mL; Eli Lilly, Indianapolis, IN, USA) at a dose of 0.5 U insulin/kg body weight was intraperitoneally administered after 4-hour fasting; blood glucose levels were measured at 0, 30, 60, 90, and 120 minutes after insulin administration.

### Body Fat Composition

Mice were anesthetized with isoflurane, immobilized in a chamber, and scanned using La Theta (LCT-100M) experimental animal CT system (Hitachi Aloka Medical, Tokyo, Japan). Contiguous 2 mm slice images from the top of the diaphragm to the base of the tail were collected for quantitative analysis of fat mass and lean body mass (visceral mass without visceral fat mass and subcutaneous fat mass) using La Theta 1.00 software as previously reported (26, 31, 33).

### *In-Vivo* ^111^In-Exendin-4 SPECT/CT

The ^111^In-exendin-4 probe [Lys^12^(^111^In-BnDTPA-Ahx)]exendin-4 was synthesized as previously reported (16). [Lys^12^(^111^In-BnDTPA-Ahx)]exendin-4 (3.0 MBq/mouse) was injected *via* the tail vein; SPECT/CT scan was performed using Triumph LabPET12/SPECT4/CT (TriFoil Imaging Inc., Chatsworth, CA, USA) as previously described (17, 18, 20). The cumulative sum of the pancreatic SPECT values was analyzed using Amira software, version 5.6.0 (FEI Visualization Sciences Group, Düsseldorf, Germany). As described in previous reports (17, 18, 20), the region of interest (ROI) was analyzed using images ranging from lower limit of the lungs to upper limit of the bladder, in which each voxel represented a 0.9-mm cube. In order to analyze only pancreatic uptake, a renal ROI was determined from outlining the kidneys on a CT image and dilating the renal ROIs by 2.7mm. This region was then excluded from the abdominal space image volume so that the remaining uptake in the abdominal space can be counted as that from pancreas. This method for defining pancreatic uptake was previously validated and showed that *in-vivo* ^111^In-exendin-4 SPECT/CT uptake was highly correlated to *ex-vivo* pancreatic uptake measured by curiemeter (17). The pancreatic uptake value per injected dose of the probe (percentage of injected dose/1 g; %ID/g) was calculated as previously reported (17, 20).

### Immunohistochemical Quantification of BCM

The mice were sacrificed by cervical dislocation; the pancreas was immediately resected. The tissue was immediately fixed in 10% formalin at 4°C. For BCM analysis, 10 sets of serial formalin-fixed paraffin-embedded sections (4 mm per section; 100 mm between each set) were stained with rabbit polyclonal anti-insulin antibody (1:100; catalog no. sc-9168; Santa Cruz Biotechnology, Santa Cruz, CA, USA) as primary antibody; Alexa Fluor 488 goat anti-rabbit antibody (1:200; catalog no. A-11008; Thermo Fisher Scientific, Waltham, MA, USA) was used as a secondary antibody, as previously reported (18, 20). Adjacent sections were also stained with hematoxylin and eosin. The pancreatic sections were analyzed using a fluorescence microscope (BZ-X700; Keyence, Osaka, Japan). BCM was calculated from histological sections using the following formula: (insulin-positive area/whole pancreas area) × pancreas weight (mg) (18–20).

### Immunohistochemical Analysis of Proliferation and Apoptosis

To evaluate β-cell proliferation, 10% formalin-fixed paraffin-embedded sections from each mouse were immunostained with guinea pig anti-insulin antibody (1:100; catalog no. ab7842; Abcam, Cambridge, MA) and rabbit anti-Ki67 antibody (1:100; catalog no. ab15580; Abcam) as primary antibodies. Alexa Fluor 488 goat anti-guinea pig antibody (1:200; catalog no. ab150185; Abcam) and Alexa Fluor 546 goat anti-rabbit antibody (1:200; catalog no. A11035; Thermo Fisher Scientific), respectively, were used as secondary antibodies. For visualization of nuclei, 4’,6-Diamido-2-phenylindole (DAPI) (Prolong Gold antifade reagent with DAPI; Invitrogen, Carlsbad, CA, USA) was used. To evaluate β-cell apoptosis, the DeadEnd™ Fluorometric TUNEL System (Promega, Madison, Wisconsin, USA) was combined with insulin and DAPI immunostaining. The ratio of insulin/Ki67/DAPI co-positive cells to total insulin-positive cells as a measure of proliferation and the ratio of insulin/TUNEL/DAPI co-positive cells to total insulin-positive cells as a measure of apoptosis were calculated for 50 islets in each mouse, as previously reported (20).

### Statistical Analysis

All data are presented as dot plots and/or mean ± SEM. Statistical analyses were performed using Student’s t-test or one way analysis of variance with the Tukey-Kramer multiple comparison tests using SPSS Statistics 26.0 software (IBM, Armonk, NY, USA). *P* values < 0.05 were considered statistically significant.

## Results

### GIP Deficiency Induces Poorer Weight Gain and Lower Non-Fasting Insulin Level Under HFD Conditions

**Figure 1:**
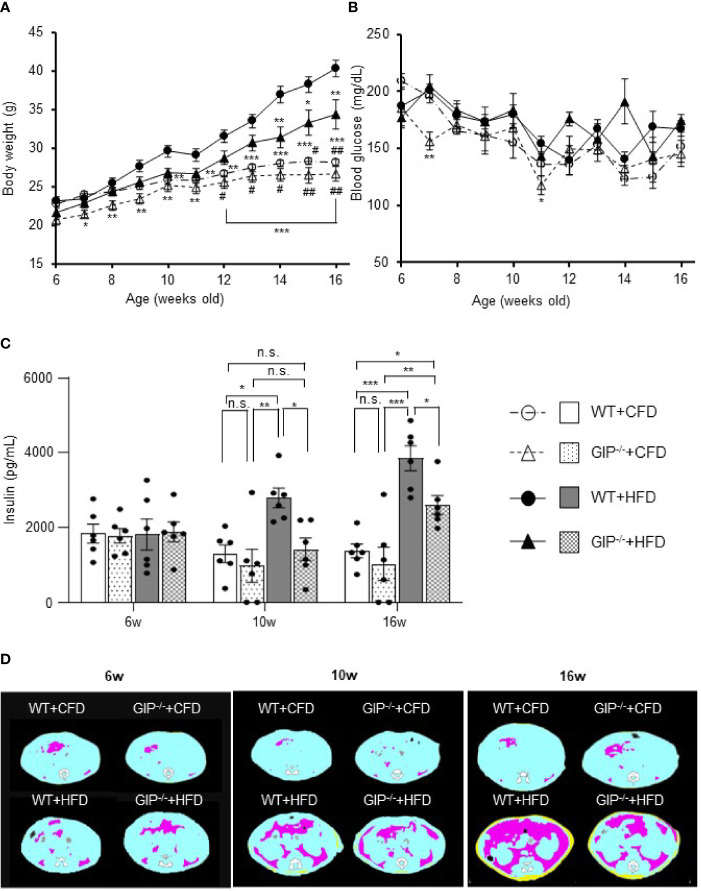
**(A)** Body weight, **(B)** non-fasting blood glucose levels, **(C)** non-fasting insulin levels during the observation period. **(D)** Representative axial computed tomography images of body fat composition at 16 weeks of age in each group. Yellow, pink, and blue areas represent subcutaneous fat, visceral fat, and lean body mass, respectively. Wild-type mice fed control-fat diet (WT+CFD) are indicated by white circles with dot-dashes **(A, B)** and white bars with solid borders **(C)** (n = 6). GIP-knockout mice fed control-fat diet (GIP^-/-^+CFD) are indicated by white triangles with dot dash **(A, B)** and dotted bars with solid borders **(C)** (n = 6). WT mice fed high-fat diet (WT+HFD), are indicated by black circles with solid dashes **(A, B)** and gray bars **(C)** (n = 6). GIP^-/-^ mice fed high-fat diet (GIP^-/-^+HFD) are indicated by black triangles with solid dashes **(A, B)** and checkerboard bars **(C)** (n = 6). *P* values are expressed as follows: **(A, B)**: *P < 0.05, **P < 0.01, ***P < 0.001 vs. WT+HFD (one way ANOVA with Tukey’s test). *^#^P* < 0.05, *^##^P* < 0.01 vs. GIP^-/-^+HFD (one way ANOVA with Tukey’s test). **(C)**: *P < 0.05, **P < 0.01, ***P < 0.001 (one way ANOVA with Tukey’s test). n.s., no statistical significance.

GIP^-/-^ and WT mice were fed with HFD or CFD from 6 weeks to 16 weeks of age. At 6 weeks of age, no significant difference in body weight was detected among the four groups. Body weight in WT+HFD mice was 14.6% and 43.0% higher than that in WT+CFD mice at 10 and 16 weeks of age (**Figure 1A**). Body weight in GIP^-/-^+HFD mice at 16 weeks of age was 21.9% higher than that in WT+CFD mice, while no significant difference in body weight was observed at 10 weeks (**Figure 1A**). No significant difference in body weight was shown between GIP^-/-^+HFD and WT+HFD mice at 10 weeks of age. At 16 weeks of age, body weight was 14.7% lower in GIP^-/-^+HFD mice as compared with that in WT+HFD mice (**Figure 1A**). No significant difference in non-fasting blood glucose level was observed among the four groups from 6 to 16 weeks of age (**Figure 1B**). There was no significant difference in non-fasting plasma insulin level among the four groups at 6 weeks of age (**Figure 1C**). Non-fasting insulin levels were 114.3% and 181.2% higher in WT+HFD mice than in WT+CFD mice at 10 and 16 weeks of age (**Figure 1C**). No significant difference in non-fasting plasma insulin level was observed between GIP^-/-^+HFD and WT+CFD mice at 10 weeks of age; however, non-fasting plasma insulin levels were 37.9% higher in GIP^-/-^+HFD mice than in WT+CFD mice at 16 weeks of age (**Figure 1C**). Non-fasting insulin levels were 49.2% and 32.5% lower in GIP^-/-^+HFD mice, respectively, than in WT+HFD mice at 10 and 16 weeks of age (**Figure 1C**). No significant difference in non-fasting total GIP level was found between WT+HFD and WT+CFD mice at 6 weeks of age (**Figure S1B**). At 10 and 16 weeks of age, non-fasting total GIP levels were 68.5% and 152.0% elevated in WT+HFD mice compared with those in WT+CFD mice (**Figure S1B**). Non-fasting total GIP levels in GIP^-/-^+HFD or GIP^-/-^+CFD mice were undetectable at 6 to 16 weeks of age.

### GIP Deficiency Leads to Attenuate Obesity and Insulin Resistance Under HFD Conditions

No significant differences in subcutaneous or visceral fat mass were observed among the four groups at 6 weeks of age (**Figures 1D**, **S2A**). Subcutaneous fat mass in WT+HFD mice was significantly increased by 2.4- and 8.6-fold as compared to WT+CFD mice at 10 and 16 weeks of age (**Figures 1D**, **S2A**). Visceral fat mass in WT+HFD mice was also significantly increased by 2.8- and 10.6-fold as compared to WT+CFD mice at 10 and 16 weeks of age (**Figures 1D**, **S2A**). At 10 weeks of age, no significant difference in fat mass was found between GIP**^-/-^+**HFD and WT+CFD mice. However, at 16 weeks of age, subcutaneous and visceral fat mass in GIP^-/-^+HFD mice were significantly increased by 5.3- and 6.5-fold, respectively, as compared with WT+CFD mice (**Figures 1D**, **S2A**). No significant difference in fat mass was observed between GIP^-/-^+HFD and WT+HFD mice at 10 weeks of age. At 16 weeks of age, subcutaneous and visceral fat mass were decreased significantly in GIP^-/-^+HFD mice as compared with WT+HFD mice (**Figures 1D**, **S2A**). No significant difference in lean body mass was found among the four groups from 6 to 16 weeks of age (**Figure S2A**).

As for insulin sensitivity, no significant difference in ITTs was observed at 6 and 10 weeks of age among the four groups. At 16 weeks of age, the change in glucose level was significantly less in WT+HFD mice as compared with WT+CFD mice (**Figure S2B**). No significant difference in glucose change was detected between GIP^-/-^+HFD and WT+CFD mice or between GIP^-/-^+HFD and WT+HFD mice (**Figure S2B**).

### GIP Deficiency Leads to Impair Glucose Tolerance and Decrease Insulin Secretion Under HFD Conditions

**Figure 2:**
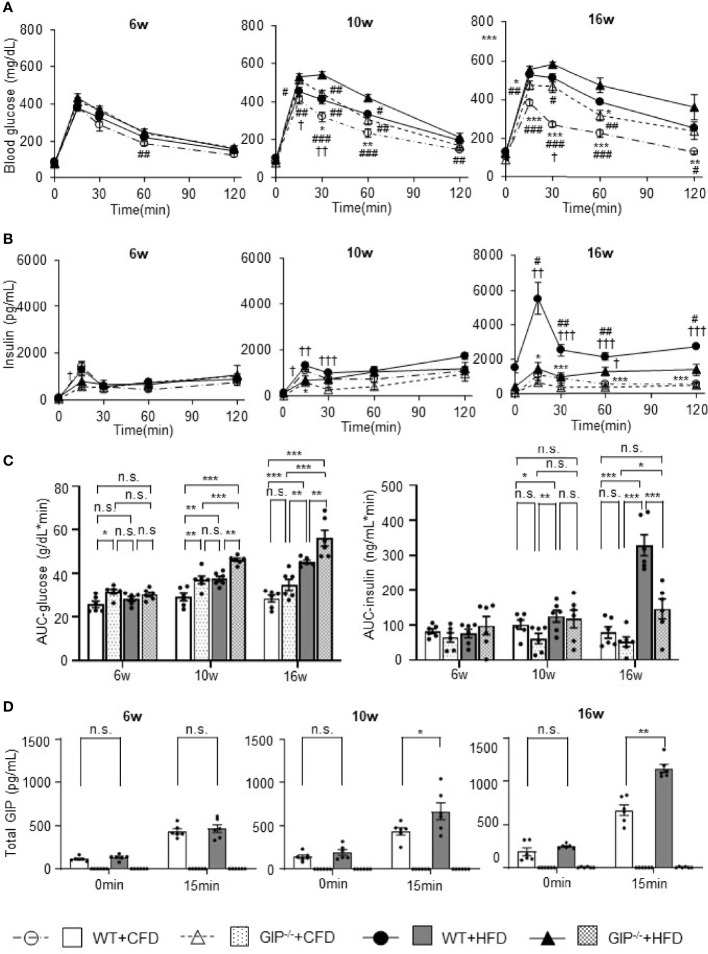
**(A)** Blood glucose levels, **(B)** insulin levels, **(C)** AUC-glucose and AUC-insulin, and **(D)** total GIP levels during oral glucose tolerance test (OGTT). WT mice fed control-fat diet (WT+CFD) are indicated by white circles with dot-dashes **(A, B)** and white bars with solid borders **(C, D)** (n = 6). GIP^-/-^ mice fed control-fat diet (GIP^-/-^+CFD) are indicated by white triangles with dot-dashes **(A, B)** and dotted bars with solid borders **(C, D)** (n = 6). WT mice fed high-fat diet (WT+HFD) are indicated by black circles with solid dashes **(A, B)** and gray bars **(C, D)** (n = 6). GIP^-/-^ mice fed high-fat diet (GIP^-/-^+HFD) are indicated by black triangles with solid dashes **(A, B)** and checkerboard bars **(C, D)** (n = 6). *P* values are expressed as follows: **(A, B)**: *P < 0.05, **P < 0.01, ***P < 0.001 vs. WT+HFD (one way ANOVA with Tukey’s test). *^#^P* < 0.05, ^##^ *P* < 0.01, ^###^ *P* < 0.001 vs. GIP^-/-^+HFD (one way ANOVA with Tukey’s test). ^†^ *P* < 0.05, ^††^ *P* < 0.01, ^†††^ *P* < 0.001 vs. GIP^-/-^+CFD (one way ANOVA with Tukey’s test). **(C)**: *P < 0.05, **P < 0.01, ***P < 0.001 (one way ANOVA with Tukey’s test). **(D)** *P < 0.05, **P < 0.01 (Student *t*-test). n.s., no statistical significance.

At 6 weeks of age, no significant differences in blood glucose, plasma insulin levels or total GIP levels during OGTT were observed among the four groups. Total GIP levels in GIP^-/-^+HFD and GIP^-/-^+CFD mice were undetectable at 6 to 16 weeks of age. Blood glucose and plasma insulin levels tended to be increased in WT+HFD mice as compared with those in WT+CFD mice at 10 and 16 weeks of age (**Figures 2A, B**). Area under the curve (AUC)-glucose and AUC-insulin during OGTT were significantly higher in WT+HFD mice as compared with those in WT+CFD mice at 10 and 16 weeks of age (**Figure 2C**). Total GIP levels in WT+HFD mice at 15 min were significantly higher than those in WT+CFD mice at 10 and 16 weeks of age (**Figure 2D**). Blood glucose and plasma insulin levels tended to be higher in GIP^-/-^+HFD mice as compared with those in WT+CFD mice at 10 and 16 weeks of age (**Figures 2A, B**). GIP^-/-^+HFD mice exhibited significantly greater AUC-glucose than WT+CFD mice at 10 and 16 weeks of age (**Figure 2C**). AUC-insulin was not significantly different between GIP^-/-^+HFD and WT+CFD mice at 10 and 16 weeks of age (**Figure 2C**). GIP^-/-^+HFD mice tended to have higher blood glucose and lower plasma insulin levels compared with those in WT+HFD mice at 10 and 16 weeks of age (**Figures 2A, B**). AUC-glucose was significantly greater in GIP^-/-^+HFD mice compared to that in WT+HFD mice at 10 and 16 weeks of age (**Figure 2C**). In addition, AUC-insulin tended to be lower in the GIP^-/-^+HFD mice than that in the WT+HFD mice at 10 weeks of age (**Figure 2C**). At 16 weeks of age, AUC-insulin was significantly lower in GIP^-/-^+HFD mice as compared with that in WT+HFD mice (**Figure 2C**).

### GIP Deficiency Attenuates BCM Expansion Under HFD Conditions As Assessed by Noninvasive Longitudinal BCM Analysis Using ^111^In-Exendin-4 SPECT/CT

**Figure 3:**
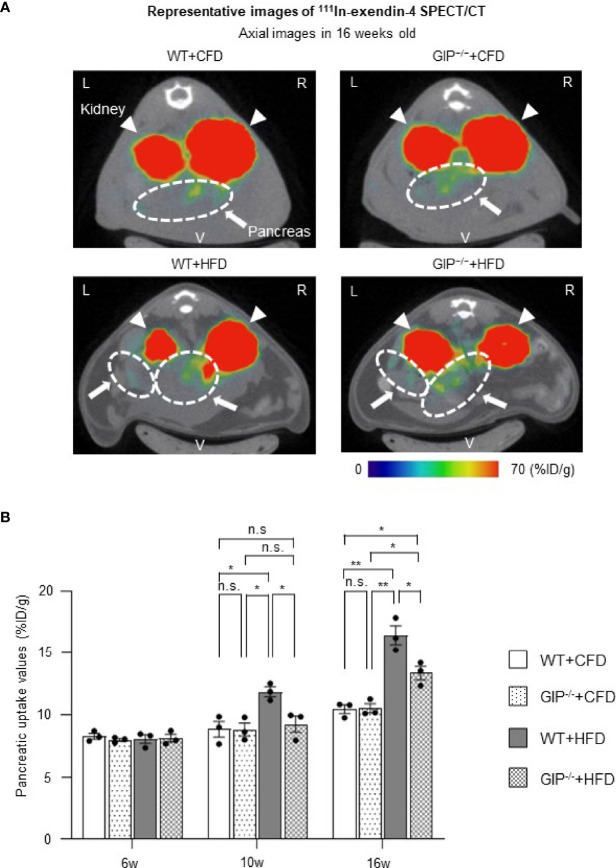
BCM changes based on ^111^In-exendin-4 SPECT/CT during the observation period. **(A)** Representative axial images of *in-vivo* ^111^In-exendin-4 SPECT/CT at 16 weeks of age in each group. The maximum to minimum intensity is indicated by color: red > orange > yellow > green > blue > black. Signals from the pancreas are indicated by white arrows and circles; signals from the kidney are indicated by white arrowheads. L, left; R, right; V, ventral. **(B)** Longitudinal changes in pancreatic uptake of *in-vivo* ^111^In-exendin-4 SPECT/CT. Pancreatic uptake values represent the percentage of injected dose/1g (%ID/g). WT mice fed control-fat diet (WT+CFD) are indicated by white bars with solid borders (n = 3). GIP^-/-^ mice fed control-fat diet (GIP^-/-^+CFD) are indicated by dotted bars with solid borders (n = 3). WT mice fed high-fat diet (WT+HFD) are indicated by gray bars (n = 3). GIP^-/-^ mice fed high-fat diet (GIP^-/-^+HFD) are indicated by checkerboard bars (n = 3). *P* values are expressed as follows: *P < 0.05, **P < 0.01 (one way ANOVA with Tukey’s test). n.s., no statistical significance.

We evaluated BCM changes using ^111^In-exendin-4 SPECT/CT at 6, 10, and 16 weeks of age. Distinct longitudinal changes in pancreatic uptake with and without HFD and/or GIP deficiency were observed (**Figures 3A, B**). Baseline pancreatic uptake values at 6 weeks of age did not differ significantly among the four groups. Pancreatic uptake values in WT+HFD mice were 34.2% and 57.1% higher than in WT+CFD mice at 10 and 16 weeks of age (**Figure 3B**). While no significant differences were observed between GIP^-/-^+HFD and WT+CFD mice at 10 weeks of age, pancreatic uptake values in GIP^-/-^+HFD mice were 27.8% higher than in WT+CFD mice at 16 weeks of age (**Figure 3B**). At 10 and 16 weeks of age, pancreatic uptake values in GIP^-/-^+HFD mice were 21.9% and 18.6% lower than in WT+HFD mice (**Figure 3B**).

### Histological Analysis Corroborates ^111^In-Exendin-4 SPECT/CT Results Indicating GIP Deficiency Attenuates BCM Expansion Under HFD Conditions

**Figure 4:**
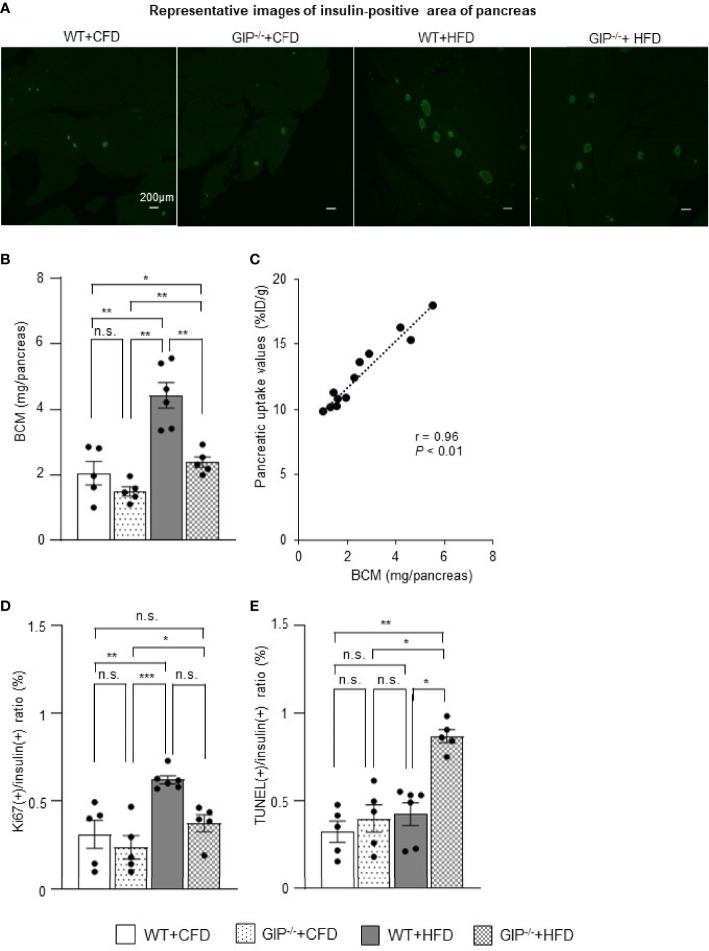
Histological quantification of BCM and immunohistochemical analysis of proliferation and apoptosis of β-cells. **(A)** Representative images of insulin-positive areas of the pancreas in each group at 16 weeks of age. **(B)** β-cell mass (BCM) was calculated as the product of the insulin-positive ratio and the pancreatic weight. **(C)** Correlation between pancreatic uptake values on ^111^In-exendin-4 SPECT/CT [percentage of injected dose/1g (%ID/g)] and BCM calculated by histological analysis in 16-week-old WT and GIP^-/-^ mice (n = 12). **(D)** Ratio of insulin/Ki67/DAPI co-positive cells to total insulin-positive cells. **(E)** Ratio of insulin/TUNEL/DAPI co-positive cells to total insulin-positive cells. WT mice fed control-fat diet (WT+CFD) are indicated by white bars with solid borders (n = 5). GIP^-/-^ mice fed control-fat diet (GIP^-/-^+CFD) are indicated by dotted bars with solid borders (n = 5). WT mice fed high-fat diet (WT+HFD) are indicated by gray bars (n = 6). GIP^-/-^ mice fed high-fat diet (GIP^-/-^+HFD) are indicated by checkerboard bars (n = 5). *P* values are expressed as follows: *P < 0.05, **P < 0.01, ***P < 0.001 (one way ANOVA with Tukey’s test). n.s., no statistical significance.

To corroborate the ^111^In-exendin-4 SPECT/CT analysis, conventional histological analysis of BCM was performed at 16 weeks of age. Histological evaluation of BCM replicated the results of the ^111^In-exendin-4 SPECT/CT analysis among all four groups (**Figures 3A, B** and **4A, B**). A significant correlation between the pancreatic uptake values measured by ^111^In-exendin-4 SPECT/CT and the histologically calculated BCM was observed in 16-week-old WT and GIP^-/-^ mice (**Figure 4C**). BCM in WT+HFD mice was 177.4% greater than that in WT+CFD mice (**Figures 4A, B**). Additionally, BCM in GIP^-/-^+HFD mice was 45.1% larger than that in WT+CFD mice (**Figures 4A, B**). On the other hand, BCM in GIP^-/-^+HFD mice was 47.7% smaller than that in WT+HFD mice (**Figures 4A, B**).

To evaluate the effects of GIP on β-cell proliferation and apoptosis, additional immunohistochemical analyses of the pancreas were performed in 16-week-old mice. WT+HFD mice had 112.9% higher ratios of insulin/Ki67 co-positive cells to total insulin-positive cells as compared with WT+CFD mice (**Figure 4D**). The ratios of insulin/Ki67 co-positive cells to total insulin-positive cells tended to be increased in GIP^-/-^+HFD mice as compared with those in WT+CFD mice, but any difference was not significant (**Figure 4D**). In addition, no significant differences in the ratios of insulin/Ki67 co-positive cells to total insulin-positive cells were found between GIP^-/-^+HFD and WT+HFD mice (**Figure 4D**). Regarding evaluation of apoptosis, no significant difference in the ratios of insulin/TUNEL co-positive cells to total insulin-positive cells was observed between WT+HFD and WT+CFD mice (**Figure 4E**). The ratios of insulin/TUNEL co-positive cells to total insulin-positive cells were 165.6% greater in GIP^-/-^+HFD mice than in WT+CFD mice (**Figure 4E**). The ratios of insulin/TUNEL co-positive cells to total insulin-positive cells were also 84.8% higher in GIP^-/-^+HFD mice than in WT+HFD mice (**Figure 4E**).

## Discussion

In the present study, ^111^In-exendin-4 SPECT/CT was utilized to reveal longitudinal BCM changes in mice with gain- and loss-of-function; obesity under HFD and GIP deficiency. Recently, the usefulness of exendin-based GLP-1R-targeted imaging such as ^111^In-exendin-4 SPECT/CT has been shown to be useful for noninvasive BCM evaluation; several studies found a significant correlation between pancreatic uptake values and BCM calculated conventionally by histological method (12, 18). In addition, pancreatic uptake values on ^111^In-exendin SPECT/CT were not affected by α-cell mass itself (12). Especially, ^111^In-exendin-4 ([Lys^12^(^111^In-BnDTPA-Ahx)]exendin-4) SPECT/CT was proved to capture the longitudinal decrease in pancreatic BCM in NOD and db/db mice (18–21). In the present study, the 16-week-old GIP^-/-^ and WT mice showed a significant correlation between pancreatic uptake values on ^111^In-exendin-4 SPECT/CT and the histological BCM regardless of the conditions of HFD feeding and GIP deficiency (**Figure 4C**). ^111^In-exendin-4 SPECT/CT was able to distinguish mice fed CFD from mice fed HFD, as their pancreatic uptake values were significantly different (WT+CFD vs. WT+HFD). Additionally, the longitudinal BCM expansion in HFD-induced non-diabetic obese mice was also demonstrated by time-course changes of the pancreatic uptake values (**Figure 3B**). Thus, the ^111^In-exendin-4 SPECT/CT technique is useful for noninvasive assessment of *in-vivo* BCM changes in both the increasing and decreasing phases.

To our knowledge, this is the first study revealing longitudinal BCM changes in GIP^-/-^ mice. Moreover, the distinct changes of BCM in mice with and without GIP secretion were demonstrated using ^111^In-exendin-4 SPECT/CT. The pancreatic uptake values of GIP^-/-^+CFD mice were similar to those of WT+CFD mice during the observation period (**Figure 3B**). Under HFD conditions, the pancreatic uptake values of GIP^-/-^+HFD mice were higher than those of WT+CFD mice at 16 weeks of age but not at 10 weeks of age (**Figure 3B**). In contrast, the pancreatic uptake values were significantly lower in GIP^-/-^+HFD mice than those in WT+HFD mice at 10 and 16 weeks (**Figure 3B**). Thus, pancreatic uptake values measured by ^111^In-exendin-4 SPECT/CT replicated the changes in BCM measured by conventional histological methods (**Figures 3A, B** and **4A, B**). In addition, the BCM results at 16 weeks of age are consistent with the results of a previous cross-sectional study (31) in which no longitudinal BCM data was reported.

Of note, in this study, the changes in the pancreatic uptake values measured by ^111^In-exendin-4 SPECT/CT analysis preceded those in fat accumulation and insulin resistance. At 10 weeks of age, the pancreatic uptake values were significantly different between GIP^-/-^+HFD and WT+HFD mice (**Figure 3B**). Similarly, non-fasting plasma insulin and total GIP levels were also significantly different between the two groups (**Figures 1C**, **S1B**). However, no significant differences in fat mass or blood glucose levels during ITT were observed between GIP^-/-^+HFD and WT+HFD mice at 10 weeks of age (**Figures S2A, B**). Therefore, the difference of the pancreatic uptake values measured by ^111^In-exendin-4 SPECT/CT at 10 weeks of age may reflect the early-stage influence of GIP secretion on BCM in response to HFD feeding.

Regarding the underlying mechanism of attenuated BCM expansion in GIP^-/-^ mice, increased β-cell apoptosis is indicated based on immunohistochemical analyses (**Figure 4E**). Such anti-apoptotic effects of GIP were consistent with the results of different *in-vivo* models (29, 30). Indeed, it is suggested the anti-apoptotic effects of GIP are mediated by the activation of CREB and the suppression of p38 mitogen-activated protein kinase as well as c-Jun N-terminal kinase (34, 35). In addition, a GIP analog has shown anti-apoptotic function and improved glucose tolerance in rat models of diabetes (36). Considering these findings, defects in GIP secretion may well have contributed to increased apoptosis of β-cells in the GIP^-/-^ mice. The present study found significantly increased β-cell apoptosis in GIP^-/-^+HFD mice as compared with that in WT+HFD mice (**Figure 4E**), while β-cell proliferation in GIP^-/-^+HFD mice tended to be reduced as compared with WT+HFD mice (**Figure 4D**). Together with the finding of no significant difference in β-cell apoptosis between GIP^-/-^+CFD and WT+CFD mice (**Figure 4E**), GIP may well be responsible for the anti-apoptotic effect on β-cells and preservation of BCM under HFD conditions.

There are several limitations to this study. Whole-body GIP^-/-^ mice were used, so it is difficult to distinguish direct and indirect effects of GIP on β-cells. Insulin hypersecretion and fat accumulation might have additively contributed to longitudinal changes of BCM in this study, and further studies using mouse models such as GIPR^-/-βCell^ mice are needed to clarify the precise mechanism of direct GIP effects on longitudinal BCM changes. In addition, although our study suggests that GIP has both proliferative and anti-apoptotic effects on β-cells in response to HFD stresses, the mice in the present study showed obesity but did not develop diabetes during the 10-week observation period. Thus, further investigation with longer-term evaluation using our GIP^-/-^ mice is required for elucidating the beneficial effects of GIP for consequential prevention of type 2 diabetes mellitus. Finally, positron emission tomography imaging with GLP-1R-targeted probes may provide better image resolution and higher sensitive information on BCM changes (9, 13).

In conclusion, this study demonstrates that ^111^In-exendin-4 SPECT/CT can capture longitudinal BCM changes in mice with HFD-induced obesity and GIP deficiency. The distinct longitudinal *in-vivo* BCM changes between GIP^-/-^ and WT mice with and without HFD feeding suggests that GIP has preferable effects on BCM in non-diabetic mice under HFD. This study demonstrates that ^111^In-exendin-4 SPECT/CT can be useful for evaluating increasing BCM and the role of GIP in BCM changes under HFD conditions.

## Data Availability Statement

The original contributions presented in the study are included in the article/**Supplementary Material**. Further inquiries can be directed to the corresponding author.

## Ethics Statement

The animal study was reviewed and approved by The animal care and use committee of Kyoto University Graduate School of Medicine.

## Author Contributions

SK and TM planned the study, researched data, contributed to discussion, wrote, reviewed and edited the manuscript. NH planned the study and contributed to discussion. HF contributed to discussion. YM, NF, KH, EI-O, TH, XL, and SY contributed to data acquisition. NI contributed to discussion, and edited the manuscript. All authors approved the final version of the manuscript.

## Funding

This study was supported by grants from the Ministry of Education, Culture, Sports, Science and Technology (MEXT), Japan Society for the Promotion of Science (JSPS) grant numbers 20H03731, 18K08475, 19K09022, 19K08977, 21K20931, Ministry of Health, Labour, and Welfare, Ministry of Agriculture, Forestry and Fisheries, Japan Diabetes Foundation, Japan Association for Diabetes Education and Care, Public Interest Incorporated Foundation, Japan Diabetes Foundation, the Japan Foundation for Applied Enzymology (Front Runner of Future Diabetes Research), MSD Life Science Foundation and Mishima Kaiun Memorial Foundation.

## Conflict of Interest

NI received joint research grants from Daiichi Sankyo Co., Ltd., Terumo Co., Ltd., and Drawbridge, Inc.; received speaker honoraria from Kowa Pharmaceutical Co., Ltd., MSD, Astellas Pharma Inc., Novo Nordisk Pharma Ltd., Ono Pharmaceutical Co., Ltd., Nippon Boehringer Ingelheim Co., Ltd., Takeda Pharmaceutical Co., Ltd., and Mitsubishi Tanabe Pharma Co., Ltd.; received scholarship grants from Kissei Pharmaceutical Co., Ltd., Sanofi, Daiichi-Sankyo Co., Ltd., Mitsubishi Tanabe Pharma Co., Ltd., Takeda Pharmaceutical Co., Ltd., Japan Tobacco Inc., Kyowa Kirin Co., Sumitomo Dainippon Pharma Co., Ltd., Astellas Pharma Inc., MSD, Eli Lilly Japan, Ono Pharmaceutical Co., Ltd., Sanwa Kagaku Kenkyusho Co. Ltd., Nippon Boehringer Ingelheim Co., Ltd., Novo Nordisk Pharma Ltd., Novartis Pharma K.K., Teijin Pharma Ltd., and Life Scan Japan Inc. NH received scholarship grant from Mitsubishi Tanabe Pharma Co., and Ono Pharmaceutical Co., Ltd.

The remaining authors declare that the research was conducted in the absence of any commercial or financial relationships that could be construed as a potential conflict of interest.

## Publisher’s Note

All claims expressed in this article are solely those of the authors and do not necessarily represent those of their affiliated organizations, or those of the publisher, the editors and the reviewers. Any product that may be evaluated in this article, or claim that may be made by its manufacturer, is not guaranteed or endorsed by the publisher.

## Abbreviations

AUC, Area under the curve; BCM, Beta cell mass; CFD, Control-fat diet; GIP, Glucose-dependent insulinotropic polypeptide/gastric inhibitory polypeptide; GIP^-/-^, GIP-knockout; GIP^-/-^+CFD, GIP-knockout mice fed control-fat diet; GIP^-/-^+HFD, GIP-knockout mice fed high-fat diet; HFD, High-fat diet; SPECT/CT, Single-photon emission computed tomography/computed tomography; WT, Wild-type; WT+CFD, Wild-type mice fed control-fat diet; WT+HFD, Wild-type mice fed high-fat diet.
