# Supplementary material for: Noninvasive Evaluation of GIP Effects on β-Cell Mass Under High-Fat Diet
Source: Front Endocrinol (Lausanne). 2022 Jul 12;13:921125. doi: 10.3389/fendo.2022.921125 (PMC9326491; doi:10.3389/fendo.2022.921125)
Supplement: Supplementary file 1 [file Presentation_1.zip › Supplementary Figures/SUPPLEMENTARY FIGURE 1-2.pptx]

## Slide 1
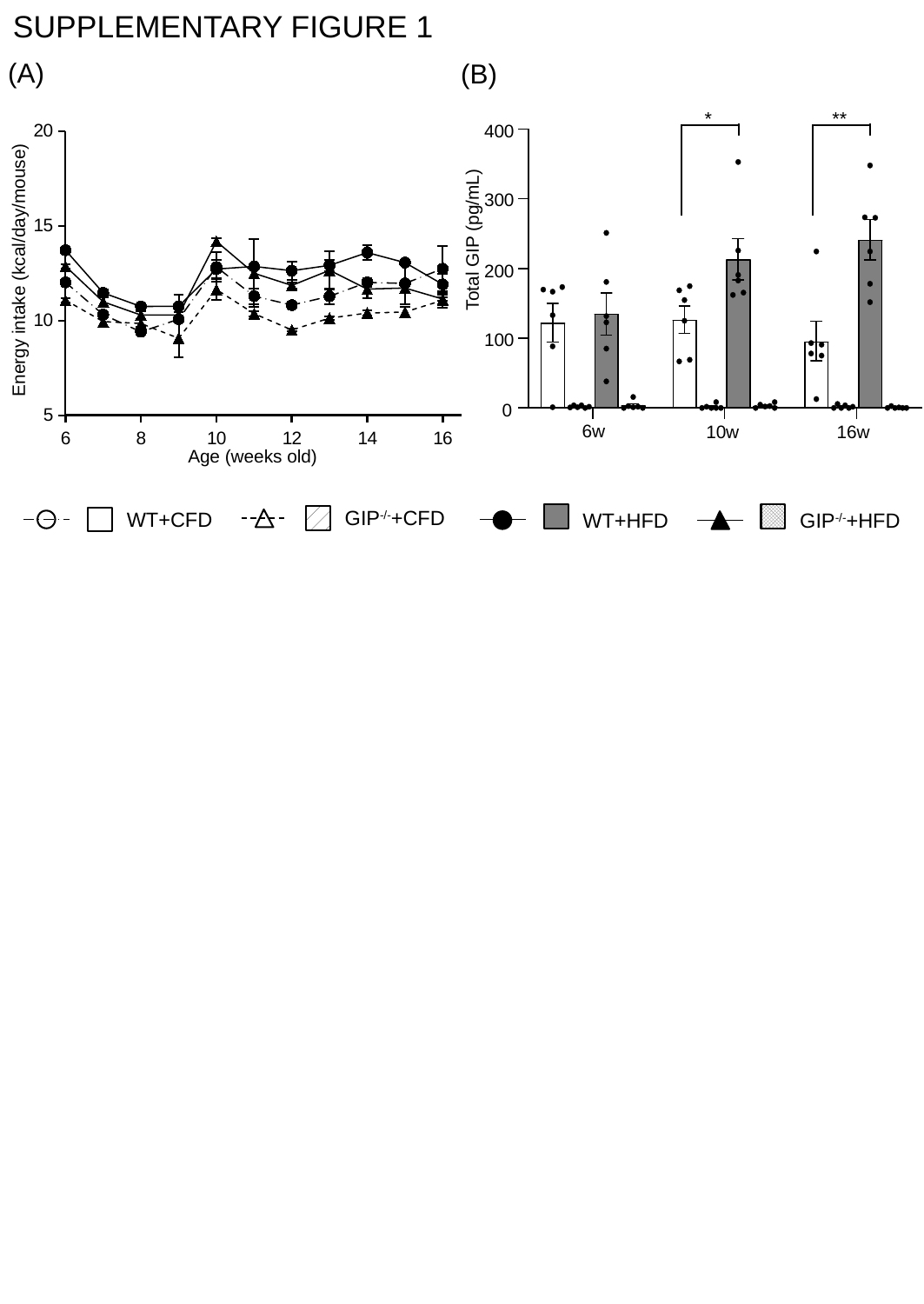

SUPPLEMENTARY FIGURE 1
(A)
(B)
*
**
### Chart
| Category | | | | |
|---|---|---|---|---|400
300
200
100
0
Total GIP (pg/mL)
Energy intake (kcal/day/mouse)
6w
16w
10w
Age (weeks old)
GIP-/-+CFD
WT+CFD
WT+HFD
GIP-/-+HFD

## Slide 2
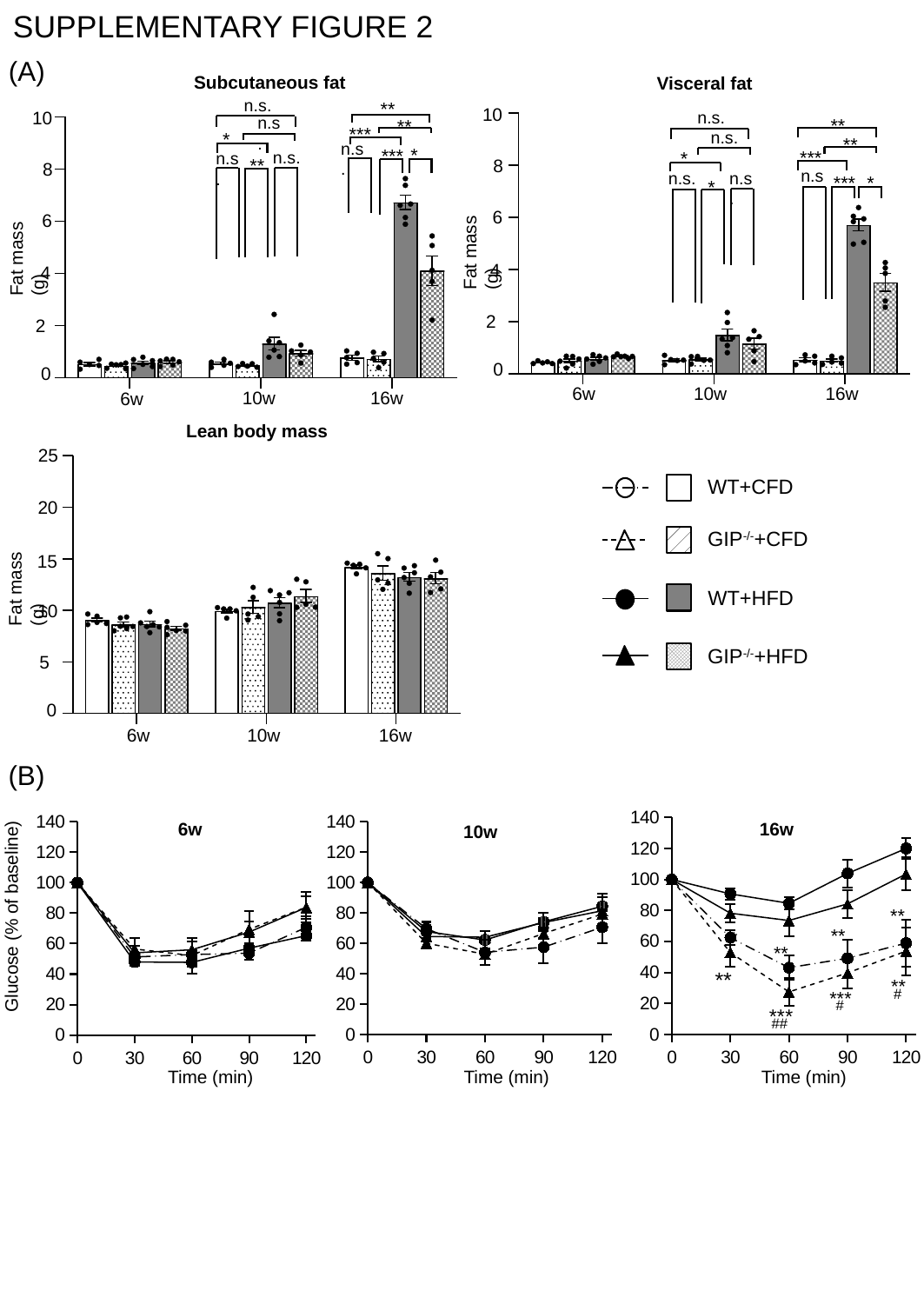

SUPPLEMENTARY FIGURE 2
(A)
Subcutaneous fat
Visceral fat
n.s.
n.s.
*
n.s.
n.s.
**
**
10
8
6
4
2
0
10
8
6
4
2
0
n.s.
n.s.
*
n.s.
n.s.
*
**
**
***
n.s.
*
***
**
***
n.s.
*
***
Fat mass (g)
Fat mass (g)
16w
6w
10w
10w
16w
6w
Lean body mass
25
20
15
10
5
0
6w
10w
16w
Fat mass (g)
WT+CFD
GIP-/-+CFD
GIP-/-+HFD
WT+HFD
(B)
### Chart
| Category | | | | |
|---|---|---|---|---|
### Chart
| Category | | | | |
|---|---|---|---|---|
### Chart
| Category | | | | |
|---|---|---|---|---|**
*
**
#
***
#
##
**
6w
16w
10w
Time (min)
Time (min)
Time (min)
Glucose (% of baseline)
**
