# Supplementary material for: Noninvasive Evaluation of GIP Effects on β-Cell Mass Under High-Fat Diet
Source: Front Endocrinol (Lausanne). 2022 Jul 12;13:921125. doi: 10.3389/fendo.2022.921125 (PMC9326491; doi:10.3389/fendo.2022.921125)
Supplement: Supplementary file 1 [file Presentation_1.zip › Supplementary Figures/SUPPLEMENTARY FIGURE LEGENDS.docx]

**SUPPLEMENTARY FIGURE LEGENDS**

**SUPPLEMENTARY FIGURE 1**

**(A)** Energy intake, **(B)** non-fasting total glucose-dependent insulinotropic polypeptide/gastric inhibitory polypeptide (GIP) levels during the observation period are shown. Wild-type mice fed control-fat diet (WT+CFD) are indicated by white circles with dot-dashes **(A)** and white bars with solid borders **(B)** (n = 6). GIP-knockout mice fed control-fat diet (GIP^-/-^+CFD) are indicated by white triangles with dot dash **(A)** and dotted bars with solid borders **(B)** (n = 6). WT mice fed high-fat diet (WT+HFD), are indicated by black circles with solid dashes **(A)** and gray bars **(B)** (n = 6). GIP^-/-^ mice fed high-fat diet (GIP^-/-^+HFD) are indicated by black triangles with solid dashes **(A)** and checkerboard bars **(B)** (n = 6). *P* values are expressed as follows: **A**: **P* < 0.05, ***P* < 0.01, ****P* < 0.001 vs. WT+HFD (one way ANOVA with Tukey’s test). *^#^P* < 0.05, *^##^P* < 0.01 vs. GIP^-/-^+HFD (one way ANOVA with Tukey’s test). **B**: **P* < 0.05, ***P* < 0.01 (Student *t*-test). n.s., no statistical significance.

**SUPPLEMENTARY FIGURE 2**

Changes in body fat composition during the observation period. **(A)** Subcutaneous fat, visceral fat, and lean body mass expressed in grams. **(B)** The glucose levels during the insulin tolerance test (ITT) represent the percentage change from fasting blood glucose levels. WT mice fed control-fat diet (WT+CFD) are indicated by white bars with solid borders **(A)** and white circles with dot-dashes **(B)**, respectively (n = 6). GIP^-/-^ mice fed control-fat diet (GIP^-/-^+CFD) are indicated by dotted bars with solid borders **(A)** and white triangles with dot-dashes **(B)** (n = 6). WT mice fed high-fat diet (WT+HFD) are indicated by gray bars **(A)** and black circles with solid dashes **(B)** (n = 6). GIP^-/-^ mice fed high-fat diet (GIP^-/-^+HFD) are indicated by checkerboard bars **(A)** and black triangles with solid dashes **(B)** (n = 6). *P* values are expressed as follows: **A**: **P* < 0.05, ***P* < 0.01, ****P* < 0.001 (one way ANOVA with Tukey’s test). **B**: **P* < 0.05, ***P* < 0.01, ****P* < 0.001 vs. WT+HFD (one way ANOVA with Tukey’s test). *^#^P* < 0.05, *^##^P* < 0.01 vs. GIP^-/-^+HFD (one way ANOVA with Tukey’s test). n.s., no statistical significance.
