# Supplementary figures and images for: Noninvasive Evaluation of GIP Effects on β-Cell Mass Under High-Fat Diet
Source: Front Endocrinol (Lausanne). 2022 Jul 12;13:921125. doi: 10.3389/fendo.2022.921125 (PMC9326491; doi:10.3389/fendo.2022.921125)

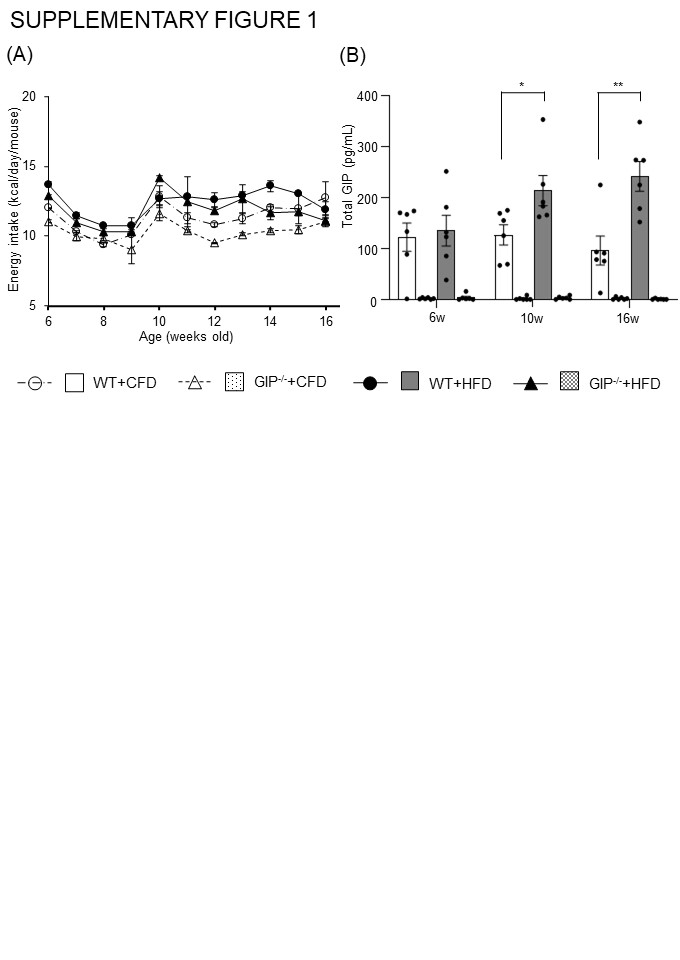

Supplement: Supplementary file 1 [file Presentation_1.zip › Supplementary Figures/SUPPLEMENTARY FIGURE 1.JPG]

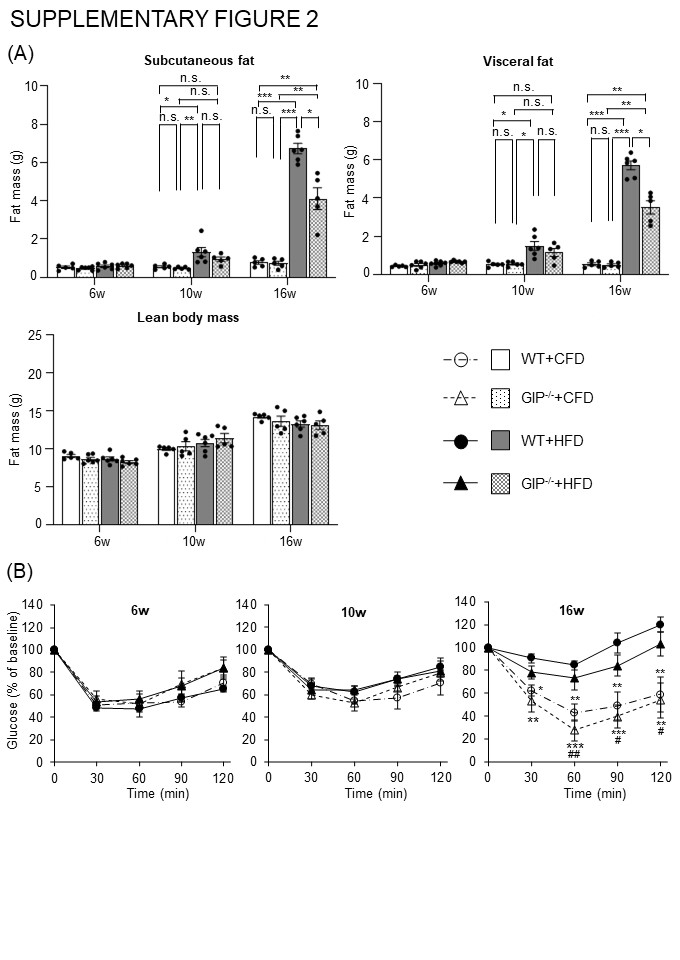

Supplement: Supplementary file 1 [file Presentation_1.zip › Supplementary Figures/SUPPLEMENTARY FIGURE 2.JPG]
